# Enhancement of Thermochemical Energy Storage by Alkali Metal Chloride Salts-Doped Ca-Based Sorbents: A Combined DFT and Experimental Study

**DOI:** 10.3390/molecules29246058

**Published:** 2024-12-23

**Authors:** Dehao Kong, Nan He, Qicheng Chen, Binjian Nie, Yingjin Zhang, Nan An, Liang Yao, Zhihui Wang

**Affiliations:** 1 School of Energy and Power Engineering, Northeast Electric Power University, Jilin 132012, China; kongdehao1997@163.com (D.K.); henan@neepu.edu.cn (N.H.); annan15932109069@163.com (N.A.); 2202100517@neepu.edu.cn (L.Y.); 1202100036@neepu.edu.cn (Z.W.); 2 Department of Engineering Science, University of Oxford, Oxford OX1 3PJ, UK; binjian.nie@eng.ox.ac.uk; 3 School of Automation Engineering, Northeast Electric Power University, Jilin 132012, China; zhangyj84@163.com

**Keywords:** calcium looping, synergistic effect, chloride salts-doping, thermochemical energy storage, DFT calculations

## Abstract

In this paper, the enhancement of thermochemical energy storage by alkali metal chloride salts-doped Ca-based sorbents is revealed by experiments and DFT calculations. The results indicate that NaCl and KCl doping increases the reaction rate and cycle stability. Compared to CaO, the conversion of NaCl-CaO and KCl-CaO after one cycle is increased by 59.1% and 61.9%, respectively. This enhancement originates from the oxygen vacancies generated by Na_2_O and K_2_O and the significantly increased surface area by CaCl_2_ as well as the sintering delay. The synergistic effect between Na_2_O, K_2_O, and CaCl_2_ increases the reaction rate of calcium-based materials. Meanwhile, the penetration of low-viscosity molten NaCl and KCl into the calcium-based materials successfully segregates the CaO grains and allows the calcium-based material to maintain the porous structure after 80 cycles, thus exhibiting a high effective conversion rate. In addition, the KCl-CaO composites show the best combined performance in terms of effective conversion and averaged thermal energy density. This work paves the way for the application of chloride salts-doped calcium-based materials.

## 1. Introduction

**Figure 1:**
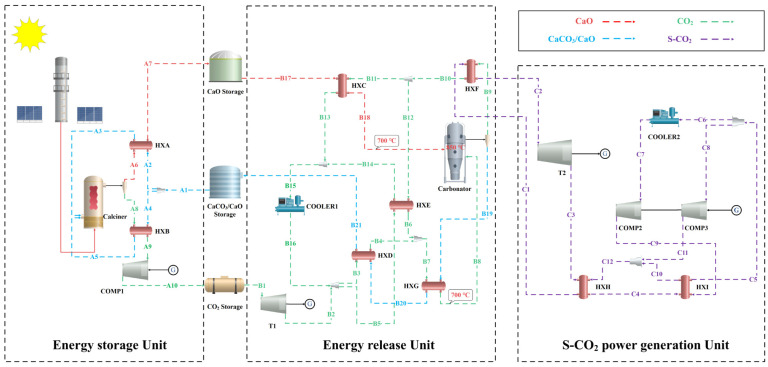
Schematic representation of the CSP-CaL system.

In recent years, Concentrated Solar Power (CSP) has gained much attention due to its high efficiency, low cost, and large scale [1,2]. However, the intermittent and fluctuating nature of solar energy fails to guarantee the long-term stable use of solar power. This problem can be addressed by thermochemical energy storage (TCES) [3,4]. In thermochemical energy storage systems, calcium-based materials are widely utilized due to their abundant availability, low cost, and high energy storage density [5,6]. Typically, CaO absorbs CO_2_ at 650–850 °C to undergo a carbonation reaction to form CaCO_3_, releasing 178 kJ/mol of heat. When the temperature exceeds 900 °C, CaCO_3_ undergoes a calcination reaction, requiring the absorption of 178 kJ/mol of heat. Therefore, calcium-based materials can store and release thermal energy through Calcium Looping (CaL) [7]. The integrated system of CSP power generation and CaL energy storage is shown in Figure 1. The CSP-CaL system has the following advantages: (i) It has a high operating temperature, which enables the implementation of a highly efficient steam cycle and thus improves the thermal efficiency of the system [8]. (ii) It has a very high energy storage density of about 3.2 GJ/m^3^ [9]. (iii) The reactants and products can be safely stored at room temperature without solidification problems [10]. Unfortunately, calcium-based material undergoes extensive sintering during the high-temperature reaction with CO_2_, which eventually leads to degradation of the storage properties [11,12].

In response to this problem, scholars have proposed numerous solutions [13,14]. For example, adding metal oxides such as MgO [15,16,17], Al_2_O_3_ [18,19], Y_2_O_3_ [20,21], and Fe_2_O_3_ [22,23] to calcium-based materials. The metal oxides could mitigate the aggregation growth and agglomeration of CaO crystals, thus inhibiting the sintering of the sorbent. However, metal oxides fail to react with CO_2_, and a thin film is formed on the metal oxide surface, limiting the reaction rate of CaL [24,25]. Recently, the doping of calcium-based materials with alkali metal salts has been suggested to increase the reaction rate [26,27,28]. For alkali metals, Reddy et al. [29] pointed out that any alkali metal loaded CaO, with the exception of Li, led to improved sorption characteristics. The absorption capacity generally increased with increasing radius of the alkali metal atom (Li < Na < K < Rb < Cs). The higher basicity of alkali metal loaded on CaO, i.e., high affinity towards CO_2_, is possibly responsible for the observed behavior. For alkali metal carbonates, Lee et al. [30] synthesized Na_2_CO_3_-CaO sorbent by the precipitation method. The results showed that Na_2_CO_3_-CaO exhibited better CO_2_ affinity above 600 °C, and its absorption/regeneration kinetics was faster than pure CaO sorbent. Luo et al. [26] proposed a novel Na_2_CO_3_-modified method to enhance the Energy Storage Density of the CaO-based materials over the close-loop CaL-TCES process, and the enhancing mechanism was investigated. The Na^+^ species that enriched on the surface of the CaO grains were in an amorphous or melting state, and they would react with CaO to form a Na-Ca solid solution over the cycles. For alkali metal sulfates, Lu et al. [31] prepared Li_2_SO_4_-CaO by wet solution impregnation method. The results showed that the addition of Li_2_SO_4_ increased the pore size of CaO and the proportion of macropores. Meanwhile, the carbonation and calcination of Li_2_SO_4_-CaO sorbent was faster than the pure CaO sorbent. Ge et al. [32] prepared MgO-supported sodium sulfate molten salt by cold-pressing method. The results showed that the formation of (Na_0.8_CaO_0.1_)_2_SO_4_ under the microscopic mechanism of competitive wetting ensured an active interface for the reaction of CaO with CO_2_ during the cycling process.

However, the studies on the doping of alkali metal chloride salts have generated different opinions. For example, Huang et al. [33] tested (Li-K) Cl-CaO and (Na-K) Cl-CaO and found that the CO_2_ uptake in the fast reaction phase was lower than pure CaO. Meanwhile, it was pointed out that the molten salt would cover the surface of the active sorbent and hinder the reaction. Similarly, Salvador et al. [34] concluded that the use of NaCl and Na_2_CO_3_ failed to improve the CO_2_ capture capacity of limestone in FBC systems. In contrast, Wu et al. [35] combined CaO with a NaCl-CaCl_2_ molten salt mixture (4:6 mass ratio) to improve CO_2_ sorption and conversion performance. Luo et al. [36] showed that KCl, NaCl, and K_2_CO_3_ boosted the carbonation property of CaO markedly, while KOH, NaOH, and Na_2_CO_3_ were detrimental to the sorbents. The synergistic effect of K^+^, Na^+^, and Cl^−^ can improve the cyclic carbonation performance of CaO. In addition, Luo et al. [37] also pointed out that NaCl modification is a promising prospect for enhancing the CO_2_ capture performance of CaO-based sorbents from flue gas. Most studies have shown that alkali metal chloride salts enhance CaL, but insights into the promoting effect at the molecular level are less well studied, thus deserving in-depth study. Meanwhile, alkali metal chloride salts are abundant and low-cost compared to sulfates and carbonates, which can be considered as a prospective dopant.

Therefore, in this paper, the role of alkali metal chloride salts NaCl and KCl in calcium looping has been deeply investigated. The promotion of calcium looping by NaCl and KCl doping is revealed by cyclic testing, SEM, XPS characterization, and DFT calculations. Meanwhile, the roles of Na_2_O, K_2_O, and CaCl_2_ in calcium looping are revealed by DFT calculations. In addition, a comprehensive evaluation of the composites based on effective conversion and averaged thermal energy density paves the way for the application of alkali metal chloride salt-doped calcium-based materials.

## 2. Results and Discussion

### 2.1. Experimental Results

The XRD analysis, particle size distribution analysis, and SEM-EDS analysis of fresh NaCl-CaO and KCl-CaO are shown in Figures S1, S2, and S3, respectively.

**Figure 2:**
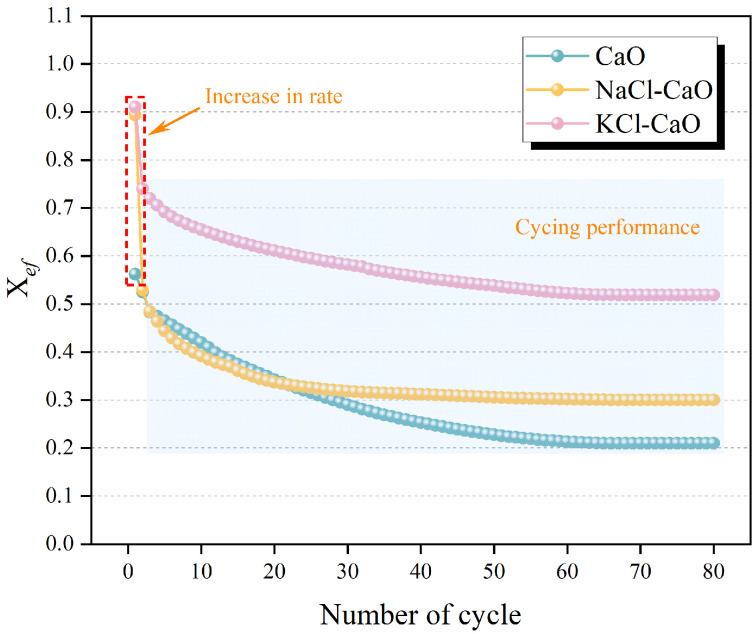
*X_ef_* of CaO, NaCl-CaO, and KCl-CaO over 80 cycles.

The *X_ef_* of CaO, NaCl-CaO, and KCl-CaO are calculated, respectively, according to Equation (5) under 80 cycles, as shown in Figure 2. It can be found that the *X_ef_* of CaO decreases gradually with the number of cycles, which is due to the low Taman temperature of CaCO_3_. The *X_ef_* of NaCl-CaO and KCl-CaO after the first cycle are 0.89 and 0.91, which are enhanced by 59.1% and 61.9%, respectively, compared to 0.562 for CaO. The *X_ef_* of NaCl-CaO and KCl-CaO after the first cycle is higher than the *X_ef_* values of MgO-doped, ZnO-doped [38], Na_2_SO_4_/MgO co-doped [32], and SiC/Mn co-doped CaO [39], indicating that the addition of NaCl and KCl increases the reaction rate between CaO and CO_2_. After 80 cycles, the *X_ef_* is 0.21 for CaO, 0.3 for NaCl-CaO, and 0.52 for KCl-CaO. The *X_ef_* of NaCl-CaO and KCl-CaO after 80 cycles is higher than the *X_ef_* values of Na_2_SO_4_-doped [28] and MgO-doped [32] CaO. It indicates that the addition of NaCl and KCl successfully inhibited the sintering of calcium-based materials, and KCl is more effective than NaCl.

**Figure 3:**
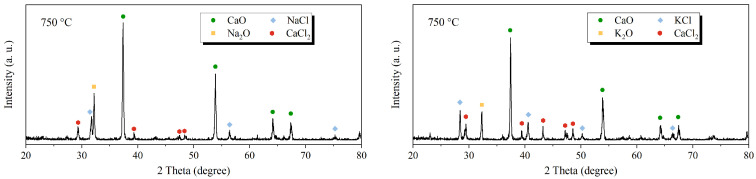
In situ XRD analysis of NaCl-CaO and KCl-CaO samples at 750 °C under an N_2_ atmosphere.

Figure 3 shows the in situ XRD analysis of NaCl-CaO and KCl-CaO samples at 750 °C under an N_2_ atmosphere. Diffraction peaks of Na_2_O (PDF: 04-003-6919) and CaCl_2_ (PDF: 01-071-5407) are detected in addition to CaO (PDF: 04-003-7161) and NaCl (PDF: 01-076-3454) in NaCl-CaO samples. Similarly, Diffraction peaks of K_2_O (PDF: 00-027-0431) and CaCl_2_ are detected in addition to CaO and KCl (PDF: 01-073-0380) in KCl-CaO samples. This implies that NaCl combines with CaO at high temperatures to form Na_2_O and CaCl_2_, while KCl combines with CaO at high temperatures to form K_2_O and CaCl_2_.

**Figure 4:**
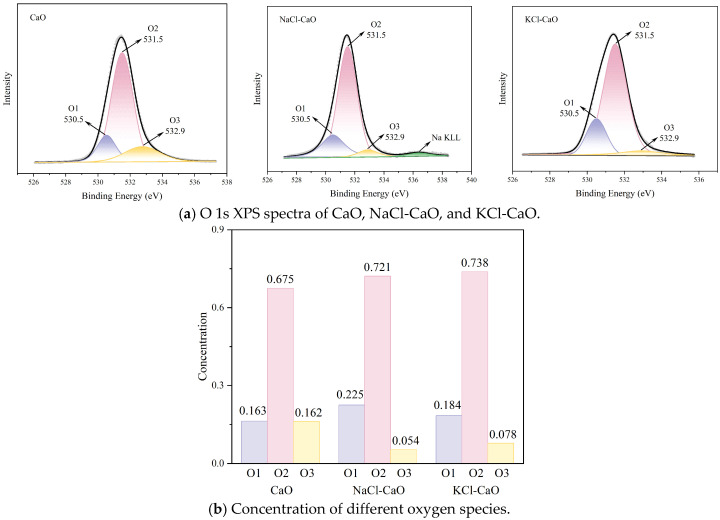
XPS spectrum for O 1s of CaO, NaCl-CaO, and KCl-CaO.

Figure 4 shows the XPS spectrum for O 1s of CaO, NaCl-CaO, and KCl-CaO. It is observed that the O 1s spectra of CaO, NaCl-CaO, and KCl-CaO can be divided into three peaks, as shown in Figure 4a. O1 represents lattice oxygen with a binding energy of 530.5 eV, O2 represents surface chemisorbed oxygen with a binding energy of 531.5 eV, and O3 represents surface oxygen-containing groups with a binding energy of 532.9 eV [40]. In particular, the concentration of chemisorbed oxygen O2 on the surface is related to the number of oxygen vacancies on the surface. A higher concentration of O2 represents a higher number of oxygen vacancies on the surface, which correlates to a higher absorption activity [41]. The concentration of O1, O2, and O3 in the samples can be evaluated based on their relative areas, as shown in Figure 4b. The results show that surface chemisorbed oxygen O2 is the main oxygen species. Among the three samples, the KCl-CaO sample has the highest concentration of oxygen vacancies, which contributes to the higher CO_2_ capture activity.

**Figure 5:**
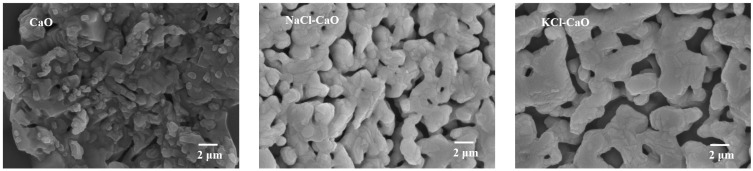
SEM images of CaO, NaCl-CaO, and KCl-CaO after cycling.

Figure 5 shows the SEM images of CaO, NaCl-CaO, and KCl-CaO after 80 cycles. It can be found that after 80 cycles, sintering of CaO occurs and the porous structure of the surface disappears, which consequently exhibits a lower *X_ef_*. For NaCl-CaO, the surface maintains a porous structure, however, the pores are small and difficult for CO_2_ transport. Notably, the KCl-CaO surface maintains a porous structure with large pores. The average viscosities of NaCl and KCl at 1123 K are calculated to be 0.95 mPa·s and 0.88 mPa·s by repeating the calculations based on Equations (7) and (8), respectively. The simulation error for NaCl molten salt is below 3%, and the average error for KCl molten salt simulation is 5.2% compared to the experimental values [42]. Due to the lower viscosity of KCl, which penetrates calcium-based materials at high temperatures and successfully separates the CaO grains, the KCl-CaO surface still maintains a porous structure with large pores after 80 cycles. In addition, the BET tests for composites are performed to verify the results obtained by TG, as shown in Figure S4.

### 2.2. Simulation Results

**Figure 6:**
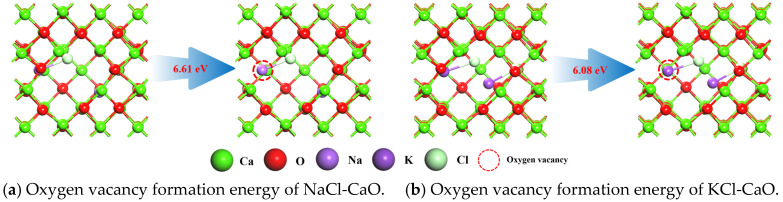
Oxygen vacancy formation energy of NaCl-CaO and KCl-CaO.

DFT calculations are performed to reveal the promotion mechanism of NaCl and KCl for Ca-based materials. The 2 × 2 × 2 supercell of CaO is established, and the energy-stabilized CaO (001) surface is selected for the study. In addition, the NaCl and KCl unit cells are placed inside CaO (001), and the surrounding CaO is deleted, thus obtaining the NaCl-CaO and KCl-CaO model, respectively. The oxygen vacancy formation energy of the CaO surface is calculated to be 6.84 eV according to Equation (9). Figure 6 shows the oxygen vacancy formation energy of NaCl-CaO and KCl-CaO. The oxygen vacancy formation energies of the NaCl-CaO surface and the KCl-CaO surface are 6.61 eV and 6.08 eV, respectively, which indicates that oxygen vacancies are easily formed on the KCl-CaO surface, consistent with the XPS analysis.

**Figure 7:**
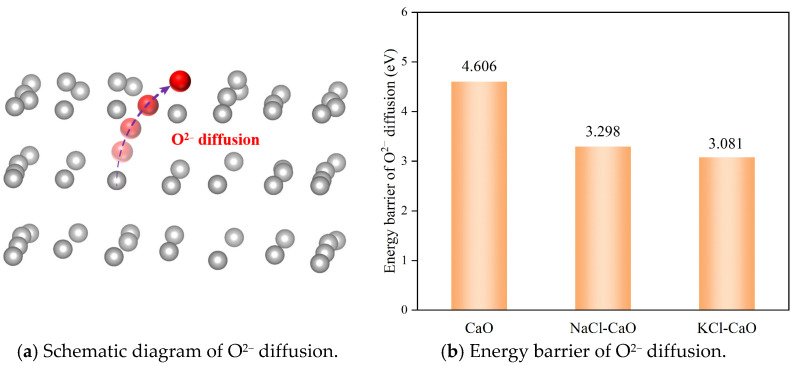
Schematic diagram and energy barrier of O^2−^ diffusion for CaO, NaCl-CaO, and KCl-CaO.

The formation of O vacancies on the surface leads to the diffusion of O^2−^ from the interior of CaO to the surface, which subsequently passes through the dense product layer and reacts with external CO_2_ to form CO_3_^2−^, as shown in Figure 7a. The rate of diffusion of O^2−^ from the interior of CaO to the surface is significant for the carbonation reaction. Figure 7b shows the energy barrier of O^2−^ diffusion, the diffusion energy barriers of O^2−^ in CaO, NaCl, and KCl are 4.606 eV, 3.298 eV, and 3.081 eV, respectively. The KCl-CaO surface has the lowest energy barrier; it indicates that the presence of KCl accelerates the diffusion of O^2−^, which consequently accelerates the combination of O^2−^ and CO_2_ to form CO_3_^2−^.

Normally, the CO_2_ adsorption process is divided into two steps. The first step is the conversion of CO_2_ from the gas-free state to the adsorbed state, and the reaction equation is as follows:

(1) $${\left({CO}_{2}\right)}_{g}↔{\left({CO}_{2}\right)}_{ads}$$

**Figure 8:**
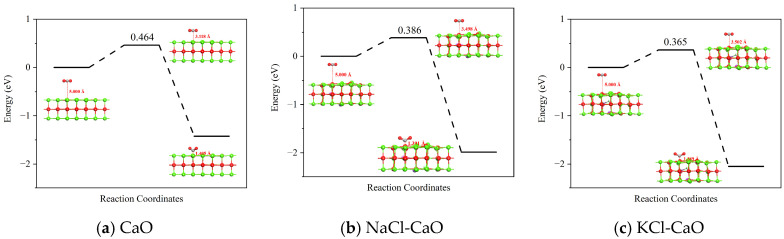
Reaction activation energy for CO_2_ adsorption on the CaO, NaCl-CaO, and KCl-CaO surfaces.

In this process, the CO_2_ adsorption capacity of the CaO, NaCl-CaO, and KCl-CaO surfaces is evaluated by the reaction activation energy, as shown in Figure 8. It can be found that the reaction activation energy of the NaCl-CaO and KCl-CaO surfaces is 0.386 eV and 0.365 eV, respectively, which are lower than 0.464 eV for the CaO surface. The decrease in reaction activation energy implies that the doping of NaCl and KCl can accelerate the conversion of CO_2_ from the gas-free state to the adsorbed state, and the enhancement of this process by KCl is superior to NaCl.

The second step of the absorption process is that CO_2_ in the adsorbed state combines with O^2−^ in CaO to form CO_3_^2−^, which then combines with Ca^2+^ in CaO to form CaCO_3_. The reaction process is as follows:

(2) $${\left(CO_{2}\right)}_{ads}+O^{2-}\to CO_{3}^{2-}$$

(3) $$Ca^{2+}+CO_{3}^{2-}\to CaCO_{3}$$

**Figure 9:**
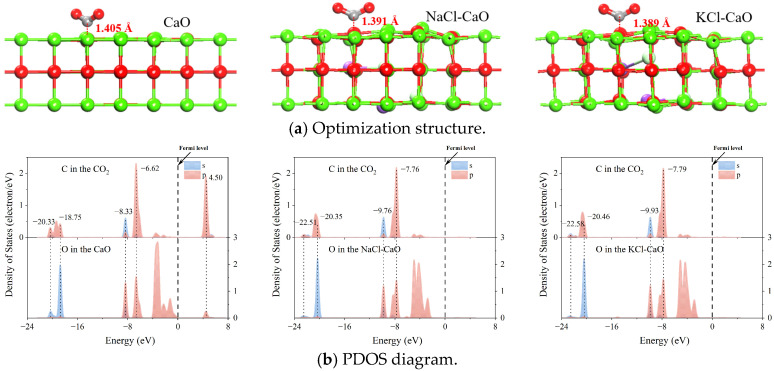
Optimization structure and PDOS for CO_2_ absorption on the CaO, NaCl-CaO, and KCl-CaO surfaces.

Figure 9a shows the optimization structure for CO_2_ absorption on the CaO, NaCl-CaO, and KCl-CaO surfaces. The interaction between the C atoms in CO_2_ and the O atoms on the surface eventually combine to form CO_3_^2−^. The optimized geometry, absorption energy, and Muliken charge transfer of CO_2_ absorption on the CaO, NaCl-CaO, and KCl-CaO surfaces are shown in Table 1. The *E_ad_* of CO_2_ on the CaO surface is −1.484 eV, and the bond length and angle of CO_2_ are 1.267/1.267 Å and 129.568°. This result is similar to the result obtained by Liu [43]. The *E_ad_* of CO_2_ on the NaCl-CaO surface is −2.699 eV, and the bond length and angle of CO_2_ are 1.262/1.284 Å and 126.664°. The *E_ad_* of CO_2_ on the KCl-CaO surface is −2.724 eV, and the bond length and angle of CO_2_ are 1.258/1.290 Å and 127.250°. Meanwhile, O atoms of the CaO surface transfer 0.644 *e* charge to CO_2_, O atoms of the NaCl-CaO surface transfer 0.693 *e* charge to CO_2_, and O atoms of the KCl-CaO surface transfer 0.704 *e* charge to CO_2_. The above-mentioned data indicate that the interaction between the O atoms of the KCl-CaO surface and the C atoms in CO_2_ is the strongest. This originates from that KCl-doped CaO produces more oxygen vacancies and accelerates O^2−^ diffusion, consistent with the experimental results.

To further demonstrate the interaction between the O atoms of the surface and the C atoms in CO_2_, the C atoms and O atoms are analyzed by the PDOS, as shown in Figure 9b. It can be found that there are five obvious resonance peaks between O atoms and C atoms in CaO, which indicates that there are obvious interactions between O atoms and C atoms, and a stable chemical bond is formed. A similar situation occurs on the NaCl-CaO and KCl-CaO surfaces. Differently, the energy resonance peaks for the *p*-orbitals of O atoms in CaO and the *sp*2 orbitals of C atoms are −6.62 eV, −8.33 eV, −18.75 eV, and −20.33 eV. For NaCl-CaO, the energy resonance peaks are −7.76 eV, −9.76 eV, −20.35 eV, and −22.51 eV. For KCl-CaO, the energy resonance peaks are −7.79 eV, −9.93 eV, −20.46 eV, and −22.58 eV. The lowest energy resonance peak between C and O atoms in KCl-CaO indicates that the presence of KCl facilitates the C-O interaction and promotes the CO_2_ absorption process.

**Figure 10:**
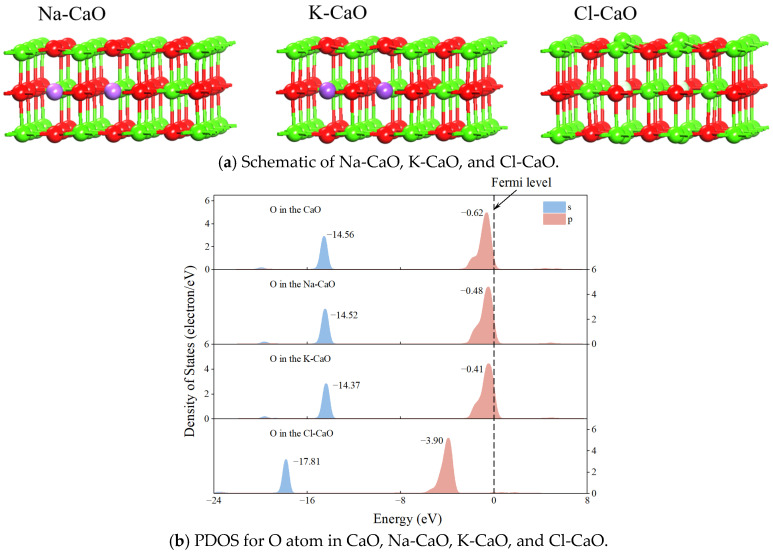
Schematic of Na-CaO, K-CaO, and Cl-CaO and PDOS for O atom in CaO, Na-CaO, K-CaO, and Cl-CaO.

Based on the XRD analysis, NaCl combines with CaO at high temperatures to form Na_2_O and CaCl_2_, while KCl combines with CaO at high temperatures to form K_2_O and CaCl_2_. Hence, Na^+^, K^+^, and Cl^−^ are doped in CaO to investigate the effect of alkali metals Na^+^, K^+^, and Cl^−^ for CaL, named Na-CaO, K-CaO, and Cl-CaO, respectively, as shown in Figure 10a. The calculated surface areas of 111.97 Å^2^ for Na-CaO and 111.99 Å^2^ for K-CaO are smaller than the surface area of 112.32 Å^2^ for CaO. This indicates that Na_2_O and K_2_O lead to a decrease in surface area, which consequently promotes the sintering of calcium-based materials [44]. For Cl-CaO, the doping of Cl causes the surface to bulge, and the surface area of Cl-CaO increases by 2.84 Å^2^ compared to CaO. This indicates that CaCl_2_ leads to an increase in surface area, which consequently delays the sintering of calcium-based materials [44].

Figure 10b shows the PDOS for the O atom in CaO, Na-CaO, K-CaO, and Cl-CaO. It is found that the energy of the *s* orbital of the O atom in CaO is −14.56 eV, and the energy of the *p* orbital is −0.62 eV. For Na-CaO and K-CaO, the *s* and *p* orbital energy of O atoms shift toward the Fermi level. Meanwhile, the oxygen vacancy formation energies on the surfaces of Na-CaO and K-CaO are calculated as 3.74 eV and 3.73 eV according to Equation (9). This indicates that the formation of Na_2_O and K_2_O promotes the sintering of the calcium-based materials while creating more oxygen vacancies on the surfaces, making the O atoms more susceptible to chemical reactions with CO_2_. For Cl-CaO, both the *s* and *p* orbital energy of the O atom shift away from the Fermi level. Meanwhile, the oxygen vacancy formation energy on the surfaces of Cl-CaO is calculated as 7.05 eV according to Equation (9). This indicates that the O atoms on the surface become more stable and the oxygen vacancies decrease. The reason for this phenomenon is that the O atoms are more electronegative than the Cl atoms, leading to stronger Ca-O interactions and more difficult detachment of O from the lattice, thus making it difficult to form oxygen vacancies. Therefore, the formation of CaCl_2_ increases the surface area and delays sintering, making the O atoms more stable, which makes it difficult to generate oxygen vacancies.

**Figure 11:**
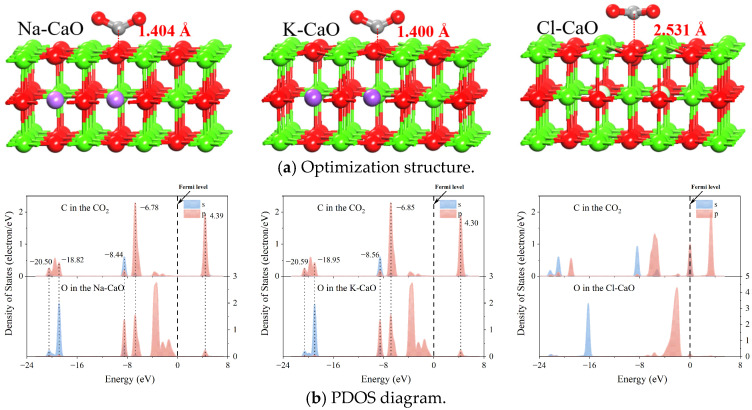
Optimization structure and PDOS for CO_2_ absorption on the Na-CaO, K-CaO, and Cl-CaO surfaces.

Figure 11a shows the optimization structure for CO_2_ absorption on the Na-CaO, K-CaO, and Cl-CaO surfaces. It is found that CO_2_ adsorbs on the Na-CaO and K-CaO surfaces to form chemical bonds with the O atoms of the surfaces. The *E_ad_* of CO_2_ on the Na-CaO surface is −1.697 eV, and the bond length and angle of CO_2_ are 1.265/1.268 Å and 129.705°. The *E_ad_* of CO_2_ on the K-CaO surface is −1.709 eV, and the bond length and angle of CO_2_ are 1.267/1.268 Å and 129.705°. For Cl-CaO, the charge on the surface is redistributed in the presence of Cl atoms. The surface O^2−^ presents a charge-deficient state and fails to transfer the charge to CO_2_, hence no chemical bond is formed between the O atom and the C atom of CO_2_. 

Figure 11b shows the PDOS between the O atoms in the Na-CaO, K-CaO, and Cl-CaO surfaces and the C atoms in CO_2_. For Na-CaO, the energy resonance peaks for the *p*-orbitals of O atoms and the *sp*2 orbitals of C atoms are 4.39 eV, −6.78 eV, −8.44 eV, −18.82 eV, and −20.50 eV. For K-CaO, the energy resonance peaks for the *p*-orbitals of O atoms and the *sp*2 orbitals of C atoms are 4.30 eV, −6.85 eV, −8.56 eV, −18.95 eV, and −20.59 eV. The presence of resonance peaks between O and C atoms indicates that there is an interaction between C and O atoms, and the interaction in K-CaO is larger than that of Na-CaO. For Cl-CaO, there is no energy resonance peak between the *p*-orbitals of O atoms and the *sp*2 orbitals of C atoms, indicating that no interaction exists between C-O atoms to form the chemical bond. Therefore, the synergistic effect between Na_2_O, K_2_O, and CaCl_2_ leads to the excellent properties of NaCl and KCl-doped CaO.

### 2.3. Thermal Energy Storage Performance and System Performance

**Figure 12:**
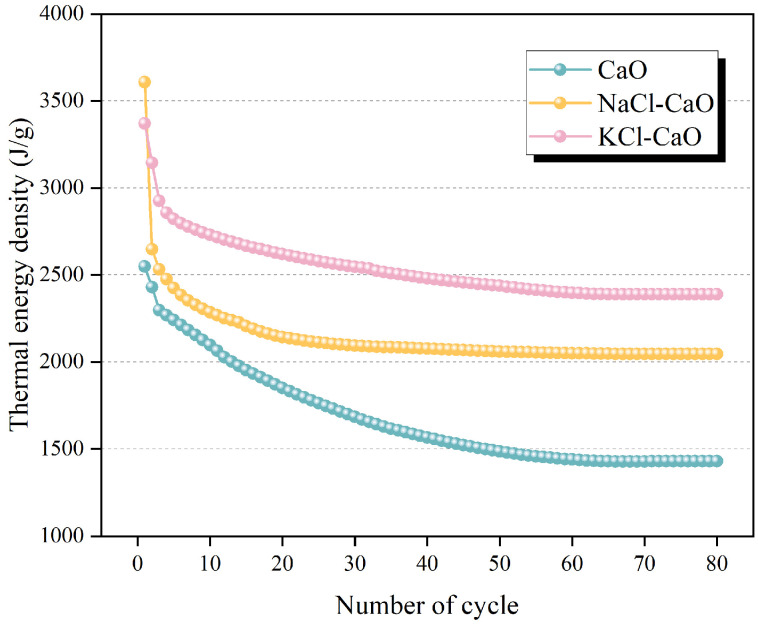
The averaged thermal energy density of CaO, NaCl-CaO, and KCl-CaO.

The averaged thermal energy density is used to evaluate the energy storage capacity of CaO, NaCl-CaO, and KCl-CaO, and the averaged thermal energy density is calculated as follows:

(4) $$Q=Q_{s}+M_{l}\times Q_{l}+M_{c}\times Q_{c}$$

where *Q_s_*, *Q_l_*, and *Q_c_* are the sensible heat, latent heat of the molten salt, and chemical reaction heat, respectively, and *M_l_* and *M_c_* are the mass fractions of the molten salt and reactive CaO in the composite, respectively. The results are shown in Figure 12. It can be found that the averaged thermal energy density of fresh CaO is 2548 J/g, and the averaged thermal energy density after 80 cycles is 1429 J/g. The averaged thermal energy density of CaO experiences a significant decay, which is similar to the trend of the effective conversion rate. For fresh NaCl-CaO and KCl-CaO, the averaged thermal energy densities are 3608 J/g and 3370 J/g, respectively. After 80 cycles, the averaged thermal energy density of NaCl-CaO and KCl-CaO are 2046 J/g and 2388 J/g. Obviously, the averaged thermal energy density of KCl-CaO is higher than those of CaO and NaCl-CaO after 80 cycles.

## 3. Experiment Procedure and Simulation Details

### 3.1. Material and Sample Preparation

The CaO (Tianjin Guangfu Technology Development Co., Ltd., Tianjin, China), NaCl (Xilong Scientific Co., Ltd., Shantou, China), and KCl (Xilong Scientific Co., Ltd., Shantou, China) used in this work are analytically pure and could be used directly without further treatment.

CaO:NaCl at a molar ratio of 5:1 and CaO:KCl at a molar ratio of 5:1 are weighed out and placed in deionized water, respectively, and stirred uniformly for 2 h. Then, the samples are dried in an oven at 120 °C for 12 h. To further remove the water from the samples, the samples are dried in a tube furnace at 500 °C under N_2_ for 2 h. Finally, the obtained samples are fully ground into powder in a mortar and filtered through a 150-mesh filter to obtain NaCl and KCl composite calcium-based materials, which are named NaCl-CaO and KCl-CaO.

### 3.2. Experimental Procedure

The carbonation and calcination processes of the composites are tested by thermogravimetry (TG, STA 449 F3, Netzsch, Germany). The experimental protocol is as follows: (a) Samples are heated from room temperature to 700 °C in a pure N_2_ atmosphere at a heating rate of 20 °C/min. (b) Carbonation process. The samples are heated from 700 °C to 850 °C in a pure CO_2_ atmosphere at a heating rate of 5 °C/min, and the heating process lasts 30 min. (c) Calcination process. The temperature is maintained at 850 °C; the atmosphere is switched to the pure N_2_ atmosphere and held for 10 min to allow the samples to calcine fully. The calcined samples are cooled down to 700 °C, and the cycle continues as described above. The cycling performance of the composites is evaluated using the effective conversion rate *X_ef_* with the following equation:

(5) $$X_{ef}=\frac{\Delta m}{A\cdot m_{0}}\cdot \frac{W_{CaO}}{W_{CO_{2}}}$$

where Δ*m* is the increase in mass during carbonation, *m*_0_ is the total mass of the composite before the carbonation reaction, *A* is the mass fraction of CaO in the composite, *W_CaO_* is the molar mass of CaO (56 g/mol), and *W_CO_*_2_ is the molar mass of CO_2_ (44 g/mol).

### 3.3. Materials Characterization

The compositions of the composites are tested by in situ X-ray diffraction (XRD) using the Anton-Paar XRK-900 reactor chamber and Bruker D8 Advance diffractometer. The experimental conditions are 40 kV and 40 mA of Cu Kα radiation (wavelength 0.15406 nm), a 2*θ* range of 20–80° in steps of 0.0131°. The microstructure and element contents are analyzed by scanning electron microscope (SEM, Regulus 8230, Hitachi, Japan) and energy dispersive X-ray spectrometry (EDS). The chemical and electronic states of the elements of the samples are analyzed by Thermo Scientific Escalab 250Xi X-ray photoelectron spectroscopy (XPS). The N_2_ adsorption–desorption isotherms and pore volume distribution of the samples are tested at 77 K using an ASAP2460 instrument following the Brunauer–Emmett–Teller (BET) and Barrett–Joyner–Halenda (BJH) methods. The particle size distribution of CaO, NaCl-CaO, and KCl-CaO are analyzed by Laser Scattering Particle Size Distribution Analyzer LA-960.

### 3.4. Simulation Details

The MD calculations are performed using the open-source software LAMMPS [45]. The simulated system consists of 5000 molecules with a 3D cubic box and periodic boundary conditions. The radius of the cutoff is 20 Å, which is less than half the length of the simulation box. All bonds are constrained with the LINCS algorithm, the cutoff for short-range non-bonded interactions is set at 1.4 nm, and long-range electrostatic interactions are solved using the Ewald method with a computational accuracy of 10^−4^. All simulated pressure states are fixed at 0.1 MPa [46,47]. The initial velocities are randomly assigned and follow Gaussian distributions, and the Newtonian equations of motion are solved using the Verlet algorithm with a time step of 1 fs [48]. The system is equilibrated in isothermal–isobaric ensemble (NPT) and canonical ensemble (NVT) for 100,000 time steps with temperature and stress damping coefficients of 100 and 1000, respectively. The results are output in micro-canonical ensemble (NVE) with a duration of 1 million time steps.

**Figure 13:**
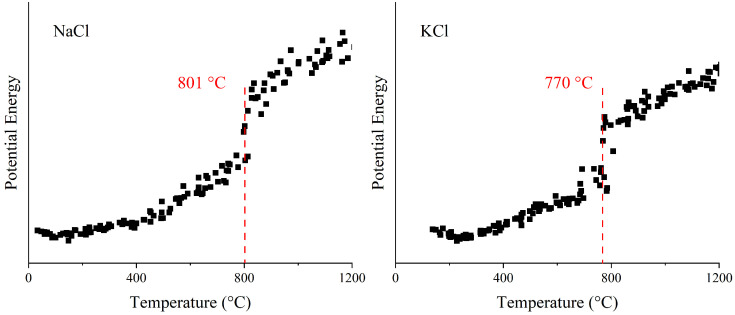
MD calculations for melting points of NaCl and KCl.

The Born–Mayer–Huggins potential function is used to calculate the interaction between molten NaCl and KCl ions with the following equation [49], the relevant parameters in the equation are shown in Table 2.

(6) $$U_{ij}=\frac{q_{i}q_{j}}{r_{ij}}+A_{ij}\exp\left(\frac{\sigma_{ij}-r_{ij}}{\rho }\right)-\frac{C_{ij}}{r_{ij}^{6}}-\frac{D_{ij}}{r_{ij}^{8}}$$

where *q_i_* and *q_j_* are the formal electrons, *q_i_* is the alkali metal ion, *q_i_* is the chloride ion, *r_ij_* is the central distance between the two ions, *σ_ij_* is the ionic radius of the crystals, *ρ* is the hardness parameter, and *C_ij_* and *D_ij_* are the Van der Waals parameters, respectively. To verify the accuracy of the potential functions, the melting points of NaCl and KCl are calculated, as shown in Figure 13. After repeating the simulation, it is found that the calculated results correspond to the theoretical values, indicating that the potential functions are correct.

To obtain the ability of NaCl and KCl to resist deformation during shear stress in the molten state, the Green–Kubo formula is used, and the shear viscosity *η* of the fluid is calculated based on the equilibrium molecular dynamics (EMD) [48,50]. The equation is as follows:

(7) $$\eta =\frac{1}{k_{B}TV}\int_{0}^{\infty }\langle P_{xy}\left(0\right)P_{xy}\left(t\right)\rangle dt$$

(8) $$P_{xy}\left(t\right)=\sum_{i=1}^{N}\left[m_{i}\upsilon_{xi}\upsilon_{yi}+\frac{1}{2}\sum_{j\neq i}x_{ij}f_{y}\left(r_{ij}\right)\right]$$

where *η* is the shear viscosity, *k_B_* is the Boltzmann constant, *T* is the temperature of the system, *V* is the volume of the system, *P_xy_*(*t*) is the pressure tensor component in the *xy* direction at *t*, *mi* is the mass of the ion *i*, *ν_xi_* and *ν_y_*_i_ are the velocity components of the particle *i* in the *x*-direction and *y*-direction, *x_ij_* is the displacement distance of the *x*-component, and *f_y_*(*r_ij_*) is the force applied from the ion *j* to the ion *i* in the *y*-component the force *f_ij_*.

The Cambridge Sequential Total Energy Package (CASTEP) code based on the Density Functional Theory (DFT) is used to reveal the mechanism of NaCl and KCl in calcium looping [51]. For accurate results, we use Generalized Gradient Approximation and Perdew Burke Enzerh (GGA-PBE) generalized functions [52,53,54,55] and set the cut-off energy to 720 eV and the k-points to 2 × 2 × 2. Meanwhile, the convergence conditions are as follows: the energy convergence criterion is less than 10^−5^ eV, the maximum force is less than 0.03 eV/Å, the maximum stress is less than 0.05 GPa, and the maximum displacement is less than 10^−3^ Å. For all surface structures used in this work, a 2 nm vacuum layer is built along the z-axis, and the bottom layer of atoms is fixed to eliminate the forces between the atoms.

Equation (9) is used to calculate the oxygen vacancy formation energy of the composite [55]. The lower energy indicates that oxygen vacancies are more likely to form on the surface of the composite.

(9) $$E_{f}^{1/2O_{2}}=E\left(reduced\right)+E\left(1/2O_{2}\right)-E\left(stoichiometric\right)$$

where *E* (*reduced*) is the energy of the surface containing O vacancies, *E* (1/2*O_2_*) is the energy of the O atom in O_2_, and *E* (*stoichiometric*) is the energy of the perfect surface.

The linear synchronous transit (LST) and quadratic synchronous transit (QST) methods are used to search for transition states [56,57]. *E_a_* is the activation energy of the reaction, *E_TS_* is the energy of the diffusion transition state, and *E_IS_* is the energy of the diffusion initial state. The smaller *E_a_* indicates that the reaction process is easier to proceed.

(10) $$E_{a}=E_{TS}-E_{IS}$$

Calculate the adsorption energy *E_ad_* by means of the following equation:

(11) $$E_{ad}=E_{CO_{2}+surface}-E_{CO_{2}}-E_{surface}$$

where *E_ad_* denotes the adsorption energy of CO_2_ on the adsorption surface, *E_CO2+surface_* denotes the total energy of the whole system after the adsorption of CO_2_, and *E_CO2_* and *E_surface_* denote the energy of the CO_2_ and the surface, respectively. For *E_ad_*, a negative value indicates exothermic adsorption, and the smaller the value, the stronger the adsorption. In addition, the partial density of states (PDOS) of the atoms is obtained with the OptaDOS program, and the Muliken charge is analyzed to evaluate the charge transfer between the atoms [58,59].

## 4. Conclusions

The enhancement of thermochemical energy storage by alkali metal chloride salts-doped Ca-based sorbents is revealed by experiments and DFT calculations. The results indicate that doping of NaCl and KCl increases the reaction rate. This enhancement derives from the synergistic effect between Na_2_O, K_2_O, and CaCl_2_. Na_2_O and K_2_O promote the sintering of calcium-based materials but increase the number of oxygen vacancies on the surface, making the O atoms more susceptible to chemical reactions with CO_2_. CaCl_2_ significantly increases the surface area and delays sintering, but it makes the O atoms more stable and less likely to generate oxygen vacancies. After the first cycle, the conversion of NaCl-CaO and KCl-CaO are 0.89 and 0.91, which are enhanced by 59.1% and 61.9%, respectively, compared to 0.562 for CaO. Meanwhile, the penetration of low-viscosity molten NaCl and KCl into the calcium-based materials successfully segregates the CaO grains, which consequently delays the sintering. After 80 cycles, the conversion of CaO is 0.21, NaCl-CaO is 0.30, and KCl-CaO is 0.52. In addition, the KCl-CaO composites are the most preferable in terms of the combination of the effective conversion rate and the averaged thermal energy density.

## Figures and Tables

**Table 1:** Optimized geometry, absorption energy, and Muliken charge transfer of CO_2_ absorption on the CaO, NaCl-CaO, and KCl-CaO surfaces.

Structure	Bond Length in CO_2_ (Å)	Bond Angle of CO_2_ (°)	*E_ad_* (eV)	Muliken Charge Transfer (*e*)
CaO	1.267/1.267	129.568	−1.484	−0.644
NaCl-CaO	1.262/1.284	126.664	−2.699	−0.693
KCl-CaO	1.258/1.290	127.250	−2.724	−0.704

**Table 2:** Potential parameters for NaCl and KCl.

Relevant Parameters	Ion Pair Type	NaCl	KCl
++	—	2.34	2.92
+−	—	2.76	3.05
−−	—	3.17	3.17
*ρ*/Å	—	0.32	0.34
	++	24.18	349.79
*C*/kcal/mol·Å^6^	+−	161.22	690.95
	−−	1669.79	1792.15
	++	11.53	345.94
*D*/kcal/mol·Å^6^	+−	200.36	1052.22
	−−	3358.46	3063.50

## Data Availability

Not applicable.

## Supplementary Materials

The following supporting information can be downloaded at: https://www.mdpi.com/article/10.3390/molecules29246058/s1, Figure S1. XRD analysis of fresh NaCl-CaO and KCl-CaO samples. Figure S2. Particle size distribution analysis of fresh CaO, NaCl-CaO and KCl-CaO samples. Figure S3. shows the SEM-EDS analysis pattern of fresh NaCl-CaO and KCl-CaO samples. Figure S4. The N_2_ adsorption-desorption isotherms and pore volume distribution of CaO, NaCl-CaO and KCl-CaO after 80 cycles. Refs. [60,61] have been cited in the Supplementary Materials.
