# Supplementary material for: Enhancement of Thermochemical Energy Storage by Alkali Metal Chloride Salts-Doped Ca-Based Sorbents: A Combined DFT and Experimental Study
Source: Molecules. 2024 Dec 23;29(24):6058. doi: 10.3390/molecules29246058 (PMC11677413; doi:10.3390/molecules29246058)
Supplement: Supplementary file 1 [file molecules-29-06058-s001.zip › molecules-3289062-supplementary.pdf]

Supplementary Material

Enhancement of thermochemical energy storage by alkali metal chloride salts-doped Ca-based sorbents: A combined DFT and experimental study

Dehao Kong [a], Qicheng Chen [a, *], Binjian Nie [b], Yingjin Zhang [c], Nan An [a], Nan He [a], Liang Yao [a], Zhihui Wang [a]

[a] *School of Energy and Power Engineering, Northeast Electric Power University, Jilin, 132012, China*

[b] *Department of Engineering Science, University of Oxford, Oxford, OX1 3PJ, UK*

[c] *School of Automation Engineering, Northeast Electric Power University, Jilin, 132012, China*

[*] *Corresponding Author: Qicheng Chen; email: chenqicheng2010@hotmail.com*

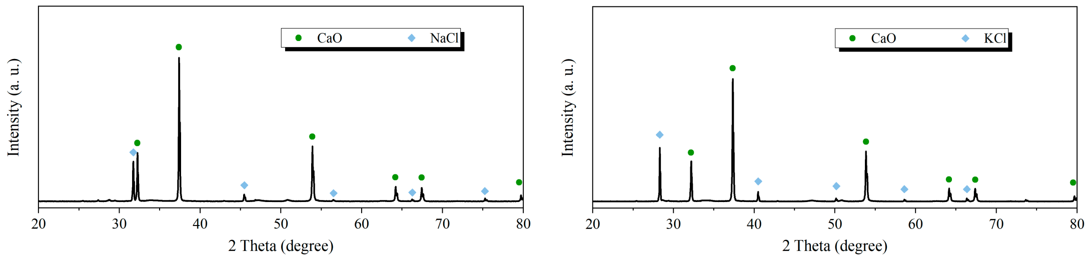

Fig. S1 XRD analysis of fresh NaCl-CaO and KCl-CaO samples.

Fig. S1 shows the XRD analysis of fresh NaCl-CaO and KCl-CaO samples. For fresh NaCl-CaO, the samples contain CaO (PDF: 04-003-7161) and NaCl (PDF: 01-076-3454). Similarly, for fresh KCl-CaO, the samples contain CaO and KCl (PDF: 01-073-0380).

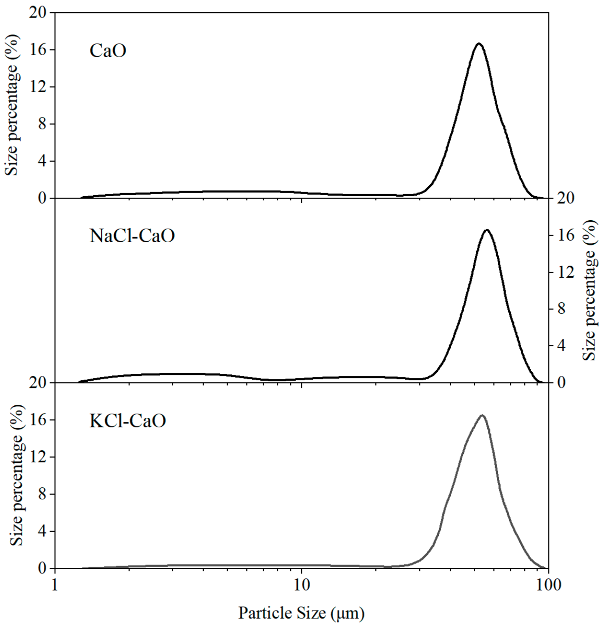

Fig. S2 Particle size distribution analysis of fresh CaO, NaCl-CaO and KCl-CaO samples.

Fig. S2 shows the particle size distribution analysis of fresh CaO, NaCl-CaO and KCl-CaO samples. The average particle sizes of NaCl-CaO and KCl-CaO are 35.12 μm and 39.32 μm, respectively, which are close to the 37.25 μm of CaO, meaning that the particle size of the

sorbent has no effect on the conversion rate.

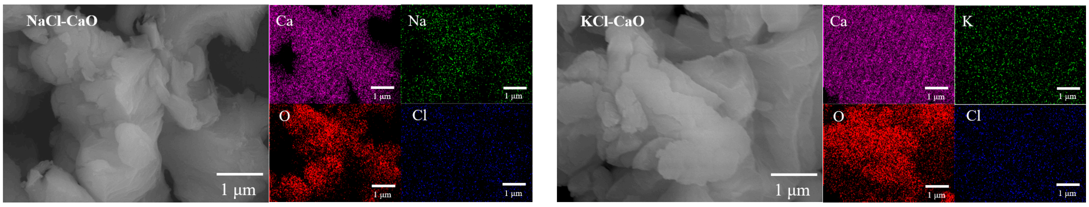

Fig. S3 SEM-EDS analysis of fresh NaCl-CaO and KCl-CaO samples.

Fig. S3 shows the SEM-EDS analysis pattern of fresh NaCl-CaO and KCl-CaO samples. For NaCl-CaO, there are Ca, O, Cl and Na in the composite, while for KCl-CaO, there are Ca, O, Cl, and K in the composite, which confirms the presence of doping elements. On the other hand, Ca, O, Cl and Na are uniformly dispersed on the surface of the composite in NaCl-CaO, and Ca, O, Cl and K are also uniformly dispersed on the surface of the composite in KCl-CaO, which proves the homogeneity of the samples.

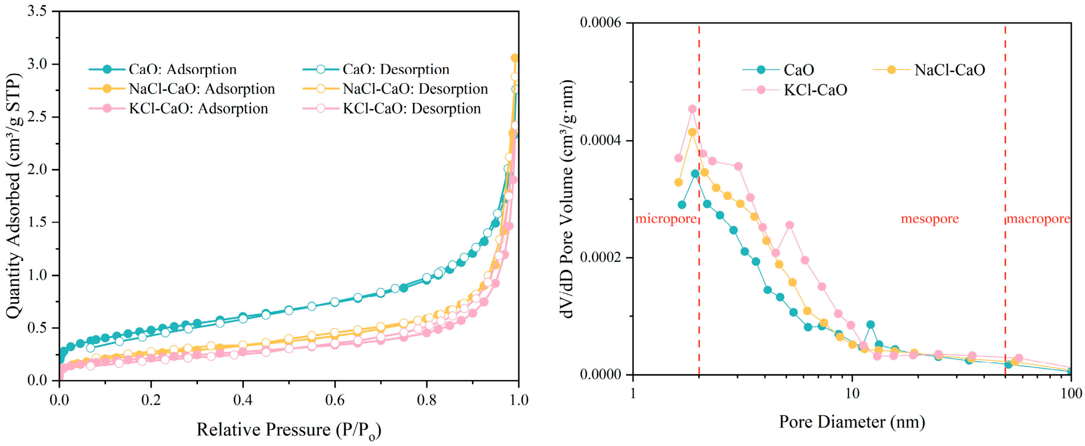

Fig. S4 The N_2 adsorption-desorption isotherms and pore volume distribution of CaO, NaCl-CaO and KCl-CaO after 80 cycles.

Fig. S4 shows the N_2 adsorption-desorption isotherms and pore volume distribution of CaO, NaCl-CaO and KCl-CaO after 80 cycles. From Fig. S4 (a), CaO, NaCl-CaO and KCl-CaO appear as reversible type II isotherms with similar shapes, which implies the mesoporous structure and monoband multilayer adsorption at high P/P_0 [1]. With the increase of P/P_0 value, after the completion of monolayer adsorption, multilayer adsorption gradually starts on the surface of the end sorbent, which shows microporous and mesoporous structures. The hysteresis loop type of H3 in the composites is determined from the stage of condensation of capillary N_2 molecules at high pressures of $0.8 \leq P/P_0 \leq 1.0$ to multilayered microporous and mesoporous sorbent configurations, indicating that non-rigid agglomerations of lamellar CaO nanoparticles

and slit-like pores [2]. The pore volume distribution of CaO, NaCl-CaO and KCl-CaO is illustrated in Fig. S4 (b). After 80 cycles, it can be found that CaO, NaCl-CaO and KCl-CaO have micro-, meso-, and macropores. Meanwhile, the BET surface area of CaO, NaCl-CaO and KCl-CaO are 0.941 m^2/g, 0.964 m^2/g and 0.972 m^2/g, respectively. Especially, KCl-CaO has more micro-, meso-, and macropores as well as BET surface area compared to other sorbents, thus maintaining a high X_{ef} after 80 cycles.

References

[1] A Lazaro, H Brouwers, G Quercia, J Geus. The properties of amorphous nano-silica synthesized by the dissolution of olivine. Chemical Engineering Journal 2012; 211-212: 112-121. https://doi.org/10.1016/j.cej.2012.09.042

[2] M Heidari, S Mousavi, F Rahmani, T Aminabhavid, M Rezakazemi. Insightful textural/morphological evaluation of cost-effective and highly sustainable Ca-Zr-O nanosorbent modified with the waste date kernel as a biomass pore-former for high-temperature CO_2 capture. Sustainable Materials and Technologies 2023; 38: e00816. https://doi.org/10.1016/j.susmat.2023.e00816
